# Dynamics of ABC Transporter P-glycoprotein in Three Conformational States

**DOI:** 10.1038/s41598-019-50578-2

**Published:** 2019-10-22

**Authors:** Noah Kopcho, Geoffrey Chang, Elizabeth A. Komives

**Affiliations:** 1 Department of Chemistry and Biochemistry, University of California, San Diego, 9500 Gilman Drive, La Jolla, CA 92093-0378 USA; 2 School of Pharmacy and Pharmaceutical Sciences, University of California, San Diego, 9500 Gilman Dr, La Jolla, AC 92093-0754 USA; 3 Department of Pharmacology, School of Medicine, University of California, San Diego, 9500 Gilman Dr, La Jolla, AC 92093-0754 USA

**Keywords:** Biophysics, Molecular biophysics, Molecular conformation

## Abstract

We used hydrogen-deuterium exchange mass spectrometry (HDX-MS) to obtain a comprehensive view of transporter dynamics (85.8% sequence coverage) occurring throughout the multidrug efflux transporter P-glycoprotein (P-gp) in three distinct conformational states: predominantly inward-facing apo P-gp, pre-hydrolytic (E552Q/E1197Q) P-gp bound to Mg^+2^-ATP, and outward-facing P-gp bound to Mg^+2^-ADP-VO_4_^−3^. Nucleotide affinity was measured with bio-layer interferometry (BLI), which yielded kinetics data that fit a two Mg^+2^-ATP binding-site model. This model has one high affinity site (3.2 ± 0.3 µM) and one low affinity site (209 ± 25 µM). Comparison of deuterium incorporation profiles revealed asymmetry between the changes undergone at the critical interfaces where nucleotide binding domains (NBDs) contact intracellular helices (ICHs). In the pre-hydrolytic state, both interfaces between ICHs and NBDs decreased exchange to similar extents relative to inward-facing P-gp. In the outward-facing state, the ICH-NBD1 interface showed decreased exchange, while the ICH-NBD2 interface showed less of an effect. The extracellular loops (ECLs) showed reduced deuterium uptake in the pre-hydrolytic state, consistent with an occluded conformation. While in the outward-facing state, increased ECL exchange corresponding to EC domain opening was observed. These findings point toward asymmetry between both NBDs, and they suggest that pre-hydrolytic P-gp occupies an occluded conformation.

## Introduction

ATP Binding Cassette (ABC) transporters comprise one of the largest families of membrane proteins. These proteins utilize ATP hydrolysis to drive substrate transport across a cell membrane. The first identified mammalian ABC transporter, P-glycoprotein, transports a diverse pool of substrate molecules unidirectionally out of cells^1^, extruding metabolites and preventing the entry of toxic molecules. P-gp has also been directly linked to numerous disease pathologies, such as tumor multidrug resistance^2^ and the progression of cerebral amyloidosis^3^.

**Figure 1:**
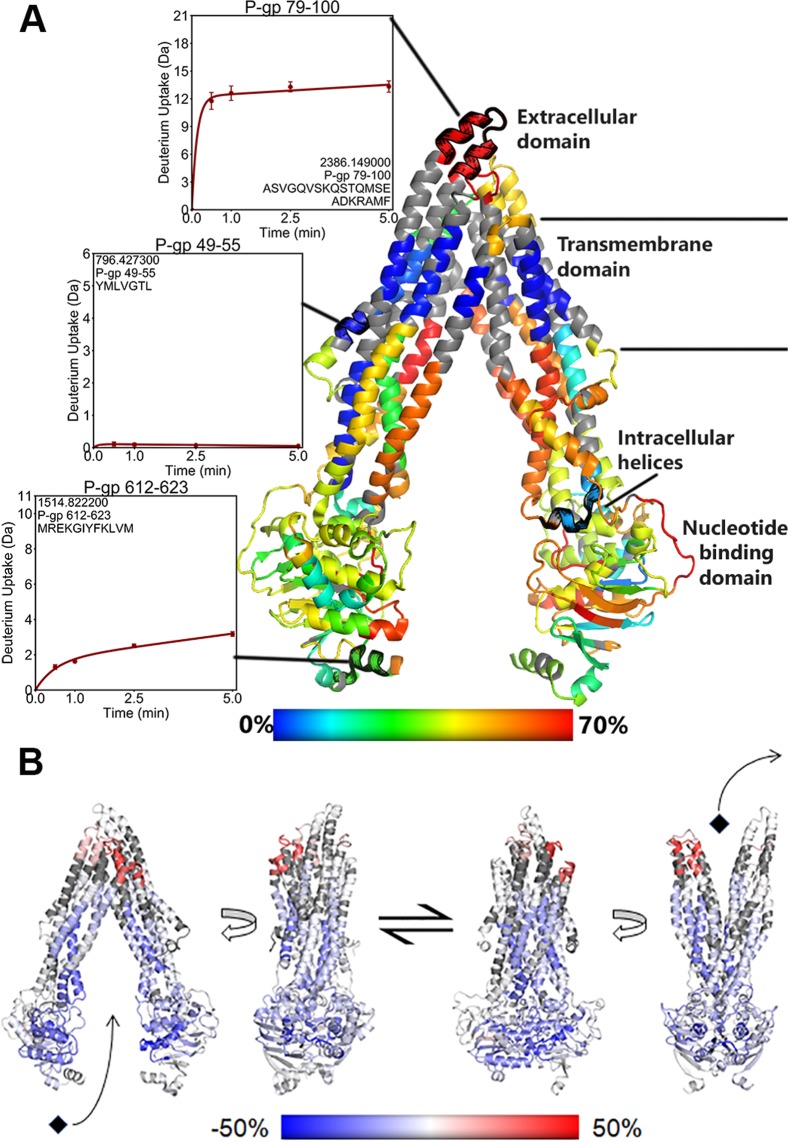
(**A)** Domain organization of P-gp. HDX-MS data have been mapped on the inward-facing crystal structure of P-gp^8^. The model is colored on a rainbow scale from lowest (blue) to highest (red) by relative deuterium uptake of apo P-gp after 5 minutes of exchange. Solvent occlusion by the detergent micelle is indicated by low exchange along the transmembrane domain. (**B)** Diagram of the conformational transition between inward and outward-facing states. A homology model based on outward-facing MsbA was used to represent outward-facing P-gp^11^. Structures are colored by difference in relative uptake between inward and outward-facing P-gp after 5 minutes of exchange. Regions with decreased exchange in the outward-facing state appear blue, while regions with greater exchange are colored red.

Structurally, P-gp is well established and consists of two homologous segments connected by a flexible linker on a single polypeptide chain (Fig. 1A). Each half of the molecule contains six transmembrane helices and one cytosolic NBD. Like many other ABC transporters, P-gp is believed to alternate between two distinct conformational states during the transport cycle: an inward-facing conformation capable of binding intracellular transport substrates, and an outward-facing conformation oriented to eject substrates across the membrane^4^. The first crystal structure of P-gp was observed in the inward-facing conformation, with the two NBDs separated from one another and a large substrate binding pocket exposed to the cytosol and inner membrane leaflet^5^. Numerous structures of inward-facing P-gp have been determined since then^6–8^.

During transport, P-gp binds to two ATP molecules and the NBDs dimerize in a head-to-tail arrangement, occluding the binding pocket from the intracellular environment. Dimerization is accompanied by ATP hydrolysis at the NBDs and the outward movement of EC helices on the opposite end of the transporter, resulting in a post-transport outward-facing conformation (Fig. 1B). These rearrangements open the binding pocket to the EC environment and result in a conformation with reduced substrate affinity, enabling substrate translocation across the plasma membrane^9^. The outward-facing conformation has been observed crystallographically in the homologous bacterial ABC transporters Sav1866^10^ and MsbA^11^. Conformational changes associated with NBD dimerization in P-gp have been validated *in vitro* using cysteine cross-linking^12^ and fluorescence resonance energy transfer (FRET)^13^ experiments. Electron paramagnetic resonance^14^ measurements with MsbA and double electron-electron resonance^15^ experiments have demonstrated increased dynamics throughout the P-gp EC domain in the outward-facing conformation.

An intermediate conformation between the inward and outward-facing states has also been observed in some transporters. Often referred to as the occluded conformation, this state represents the point at which NBDs have dimerized yet the EC domain opening has not yet occurred^16^. The existence of this conformation has been supported by experimental evidence utilizing FRET, cysteine cross-linking and cysteine accessibility to demonstrate a conformation in which NBDs were dimerized while the EC domain remained closed in MsbA^17^. The occluded conformation was later observed via crystal structures of the bacterial homologs McjD^18^ and PglK^19^, and in a cryo-EM structure of MsbA^20^.

The transport cycle of P-gp has typically been studied through the use of site-directed mutagenesis and specific ligands which arrest the molecule in various intermediate conformations. The pre-hydrolytic ATP bound state has been stabilized through the addition of non-hydrolyzable ATP analogues^21^ and mutations which inhibit ATPase activity^22^. The mutation of key catalytic residues within the NBDs (E552Q/E1197Q) was found to stabilize the ATP-bound pre-hydrolytic state by dramatically slowing P-gp catalyzed ATP hydrolysis^23^, and these combined mutations were recently used to generate a cryo-EM structure of P-gp bound to ATP^24^. When P-gp carries out ATP hydrolysis in the presence of the orthovanadate ion (VO_4_^−3^), the P-gp molecule becomes trapped in the outward-facing Mg^+^2-ADP-VO_4_^−3^-bound post-hydrolytic state^25^. Orthovanadate trapping stabilizes the hydrolytic transition state by mimicking the γ-phosphate of ATP, and has been used previously to form stable transition state complexes with ATPase enzymes^26^. We have interrogated the dynamics of P-gp in three distinct states: apo P-gp (inward-facing), Mg^+^2-ATP bound to (E552Q/E1197Q) P-gp (pre-hydrolytic), and P-gp bound to Mg^+^2-ADP-VO_4_^−3^ (post-hydrolytic, outward-facing).

Hydrogen-deuterium exchange mass spectrometry (HDX-MS) is ideal for studying protein dynamics as they occur in solution. HDX-MS exploits the phenomenon of backbone amide hydrogen-deuterium exchange to probe changes in protein dynamics and solvent accessibility^27^. When exposed to buffered D_2_O, amide hydrogens will exchange with deuterons in the same manner as they would react with protons from H_2_O. Exchange occurs more rapidly for labile protons that are exposed to solvent than for those that are confined by structural elements^28^. As a protein naturally samples a wide range of conformations in solution, every amide proton will eventually exchange with deuterium. Thus, by measuring deuterium incorporation as a function of time, a picture of regional dynamics emerges.

In recent years, HDX-MS has gained attention as a powerful modality to study membrane proteins^29–31^. Previous studies have applied this method to probe the conformational dynamics of P-gp^32^ and the *B*. *subtilis* ABC transporter BmrA^33^. Although success has been limited by low sequence coverage obtained following MS analysis (40.1% coverage in the former experiment and 67.5% for the latter), both studies noted conformational heterogeneity in the ligand-free state as well as different responses to nucleotide hydrolysis between both halves of the molecule. The data presented here were obtained following rigorous optimization of peptide separation parameters to obtain coverage of transporter regions unobserved in previous HDX-MS experiments. Our comparison of the resulting deuterium incorporation profiles reveals differences in dynamics between the three states, and it suggests that the pre-hydrolytic ATP-bound state occupies the occluded conformation.

## Results

**Figure 2:**
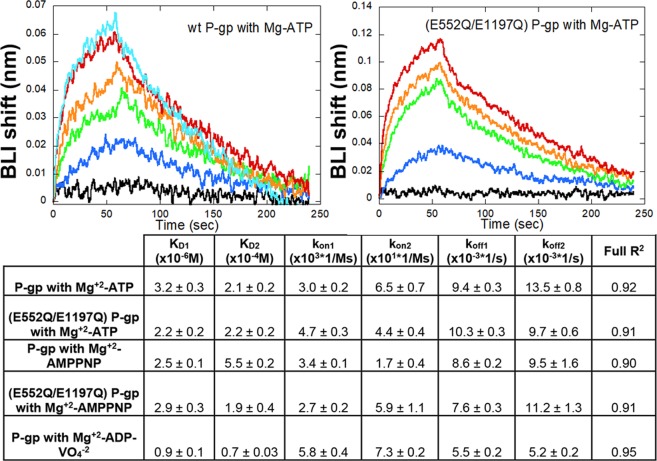
Binding kinetics resulting from BLI experiments are summarized. Both mutant and wild-type P-gp displayed similar affinities for ATP and AMPPNP. Wild-type P-gp binding to Mg^+2^-ATP in the presence of VO_4_^−3^ was approximately 10-fold tighter. Concentrations are 1.6 (black), 8 (blue), 40 (green), 200 (orange), 1000 (red) and 5000 (cyan) µM. The kinetic parameters are listed in the Table below the plots.

We carried out HDX-MS on apo P-gp, which has been shown to predominantly occupy the inward-facing conformation^15,34^, pre-hydrolytic (E552Q/E1197Q) P-gp bound to Mg^+^2-ATP^23,24^, and outward-facing P-gp stabilized by complexation with Mg^+2^-ADP-VO_4_^−3 ^^25^. To verify nucleotide binding affinities of both P-gp constructs, binding of Mg^+2^-ATP and Mg^+2^-AMPPNP were measured using BLI (Fig. 2). In each case, the best fit to the data was obtained by using a two binding-site model, which revealed one site having low micromolar affinity and another in the hundreds of micromolar range, indicating a strong asymmetry in nucleotide binding to each NBD. Affinity of wild-type P-gp for Mg^+2^-ADP-VO_4_^−3^ also fit to a two binding-site model, though it could not be determined whether the VO_4_^−3^ ion was present at both sites. The measured affinities in this case were approximately 10-fold tighter than when measured in the absence of VO_4_^−3^, also in agreement with previous findings^35^. Despite similar binding kinetics (Supp. Fig. 1), the mutant (E552Q/E1197Q) P-gp was verified as being insensitive to verapamil-stimulated ATPase activity (Supp. Fig. 2)^23^.

Our HDX-MS data covered 85.8% of the P-gp sequence in 86 different peptides (Supp. Fig. 3). Deuterium uptake was measured at time intervals up to 5 min of incubation in deuterated buffer at 25 °C, as these conditions were recently shown to reveal native state protein dynamics that occur in the μs-ms range^36^. Fractional deuterium uptake into apo P-gp was mapped onto the crystal structure of mouse P-gp^8^ providing a measure of dynamics occurring throughout the transporter (Fig. 1A). In order to contextualize differences in conformational dynamics between the inward and outward-facing states, we compared deuterium uptake between apo P-gp and outward-facing P-gp. Differences of more than 0.5 Da were interpreted as significant. The difference in uptake between the inward-facing, pre-hydrolytic and outward-facing states were mapped on models which yielded the best structural fit to our data.

### Transmembrane domain

The linker connecting both halves of P-gp has not been observed in three-dimensional structures. Contrary to the fast exchange expected for an unstructured region, deuterium uptake into the linker sequence (covered by peptides corresponding to residues 619–630, 631–658 and 659–684) was not complete even after 5 min of exchange (Supp. Fig. 4). These results suggest that the linker may either possess some degree of secondary structure, as hypothesized previously^37^, or that it contacts other surfaces of the transporter. The linker showed no measurable differences between the three experimental states of P-gp.

**Figure 3:**
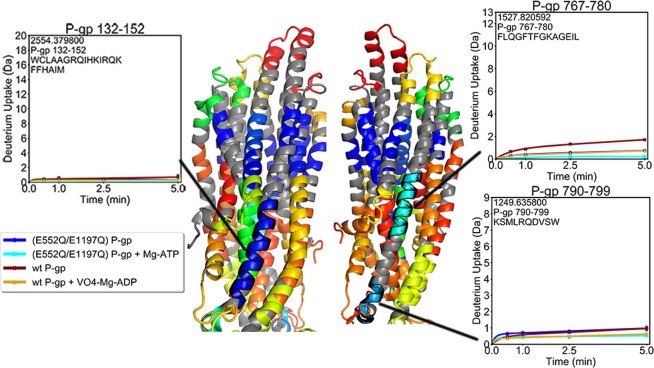
Deuterium uptake plots for regions of TM2 and TM8. These helices are centered along the sides of the transporter, and are the only TMHs which displayed low uptake outside the detergent band.

**Figure 4:**
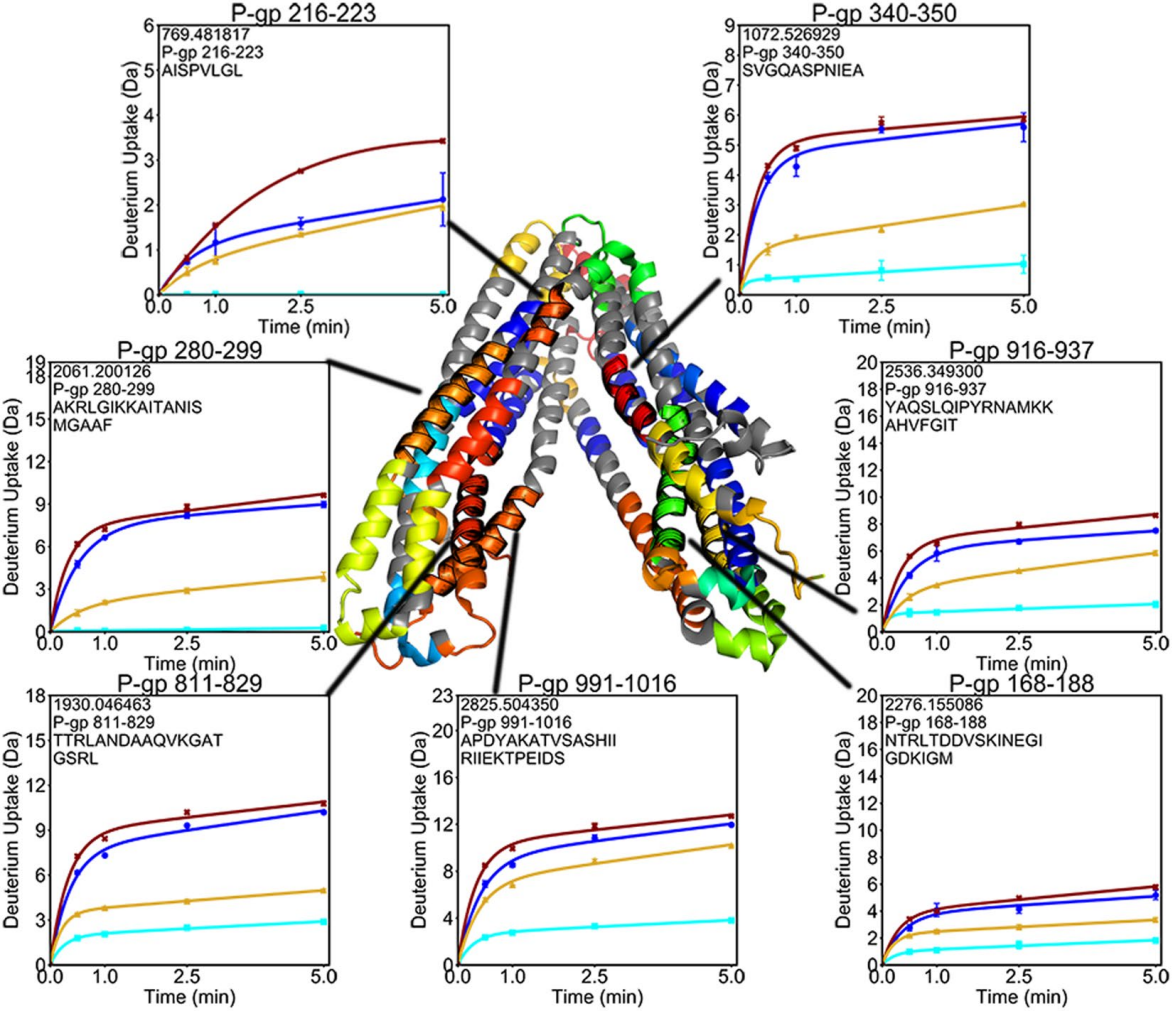
Deuterium uptake plots for the TMHs comprising the polyspecific binding pocket. All regions display increasing uptake over time, indicative of ongoing dynamic processes throughout this region. TM4 (residues 216–223) is the only region within the detergent band that exchanged with deuterium.

In all three experimental states, a band of non-exchanging amides was observed around the transmembrane helices (TMHs) which most likely marks the region of the TMHs covered by the detergent micelle (Fig. 1A). The only TM regions which showed little deuterium incorporation outside of the detergent band were found in TM2 (residues 132–152) and TM8 (residues 767–780 and 790–799), which run along the outer sides of the transporter (Fig. 3). One peptide from TM4 (residues 216–223) incorporated deuterium despite being located along the non-exchanging band (Fig. 4). This dynamic segment of TM4 had lower exchange in the (E552Q/E1197Q) mutant than in wild-type P-gp, and both the pre-hydrolytic and outward-facing states showed lower exchange compared to the apo states. On the opposite side of the transporter, TM10 (residues 858–865) is in an analogous position but exhibited the same complete protection observed for the rest of the non-exchanging band (Supp. Fig. 4). Regions of TMHs which comprise the substrate-binding cavity (residues 56–68, 168–188, 216–223, 280–299, 340–350, 811–829, 916–937, and 991–1016) showed increasing deuterium incorporation over time, indicative of slow dynamic processes. Portions of TMHs on the intracellular side of the transporter showed decreased exchange in both the pre-hydrolytic and outward-facing states, consistent with TMH bundling that accompanies NBD dimerization (Supp. Fig. 5).

### Nucleotide binding domains

**Figure 5:**
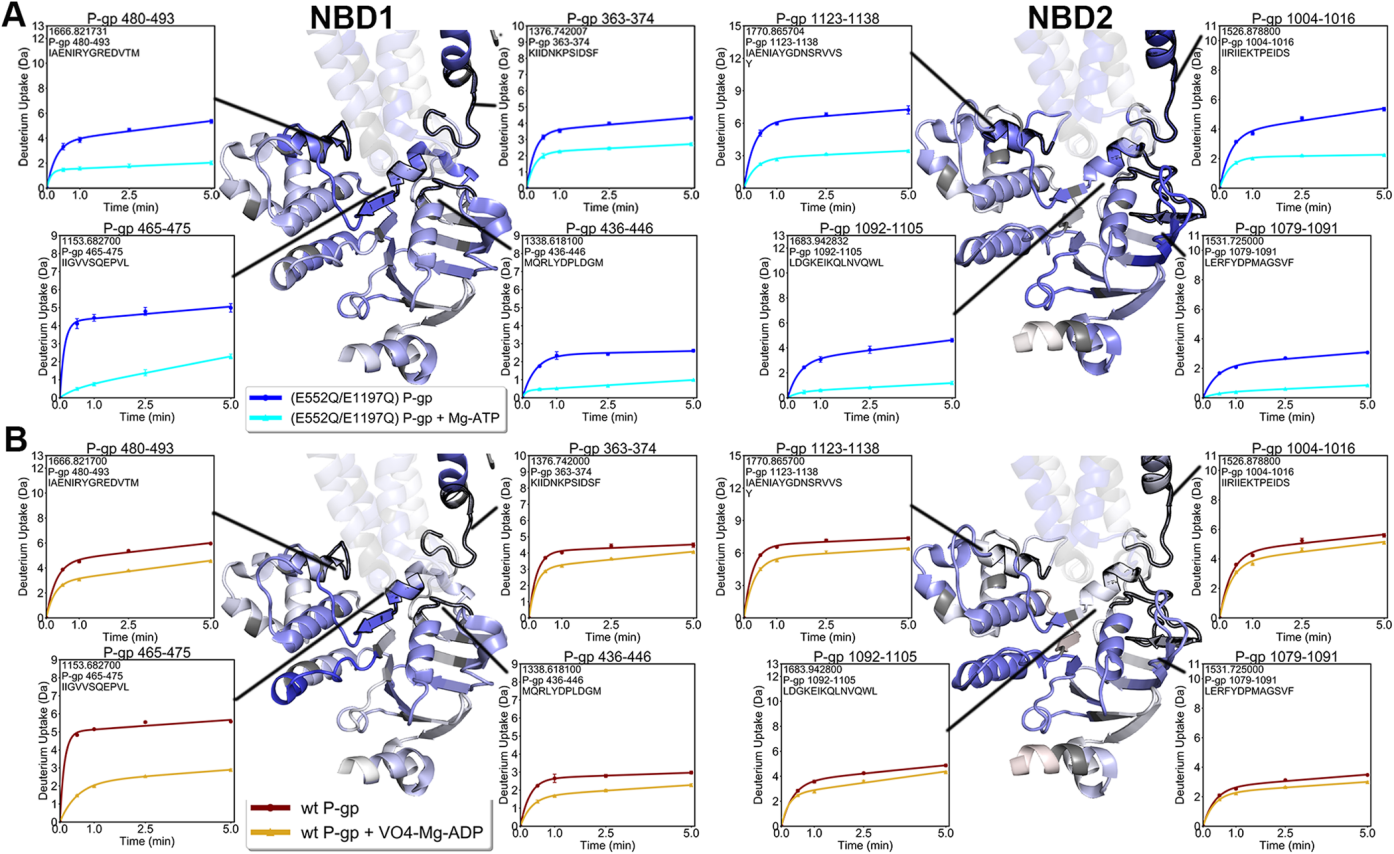
Deuterium uptake plots of the regions corresponding to NBD regions contacting ICHs. (**A)** All regions contacting ICHs from both NBDs decreased exchange in the pre-hydrolytic state. (**B)** In the outward-facing state, every region contacting ICHs on NBD1 decreased exchange by more than 0.5 Da. Only the peptide containing residues 1123–1138 showed decreased exchange in NBD2.

Within the NBDs, all conserved ATP binding motifs became more protected from exchange in both the pre-hydrolytic and outward-facing states (Supp. Fig. 6). Although previous HDX-MS of P-gp showed some evidence of EX1 kinetics within the NBDs, the phenomenon was mainly present in nanodisc-embedded P-gp and hardly observable in detergent-solublized preparations^32^. Our results are consistent with this previous work. Each NBD contacts one pair of ICHs. In the pre-hydrolytic state, all surfaces contacting ICHs in NBD1 (residues 363–374, 436–446, 465–475, and 480–493) and NBD2 (residues 1004–1016, 1079–1091, 1092–1105, 1123–1138) had decreased exchange (Fig. 5A). In the outward-facing state, all NBD1 regions contacting ICHs showed decreased exchange, while only the peptide corresponding to residues 1123–1138 decreased exchange by more than 0.5 Da in NBD2 (Fig. 5B).

**Figure 6:**
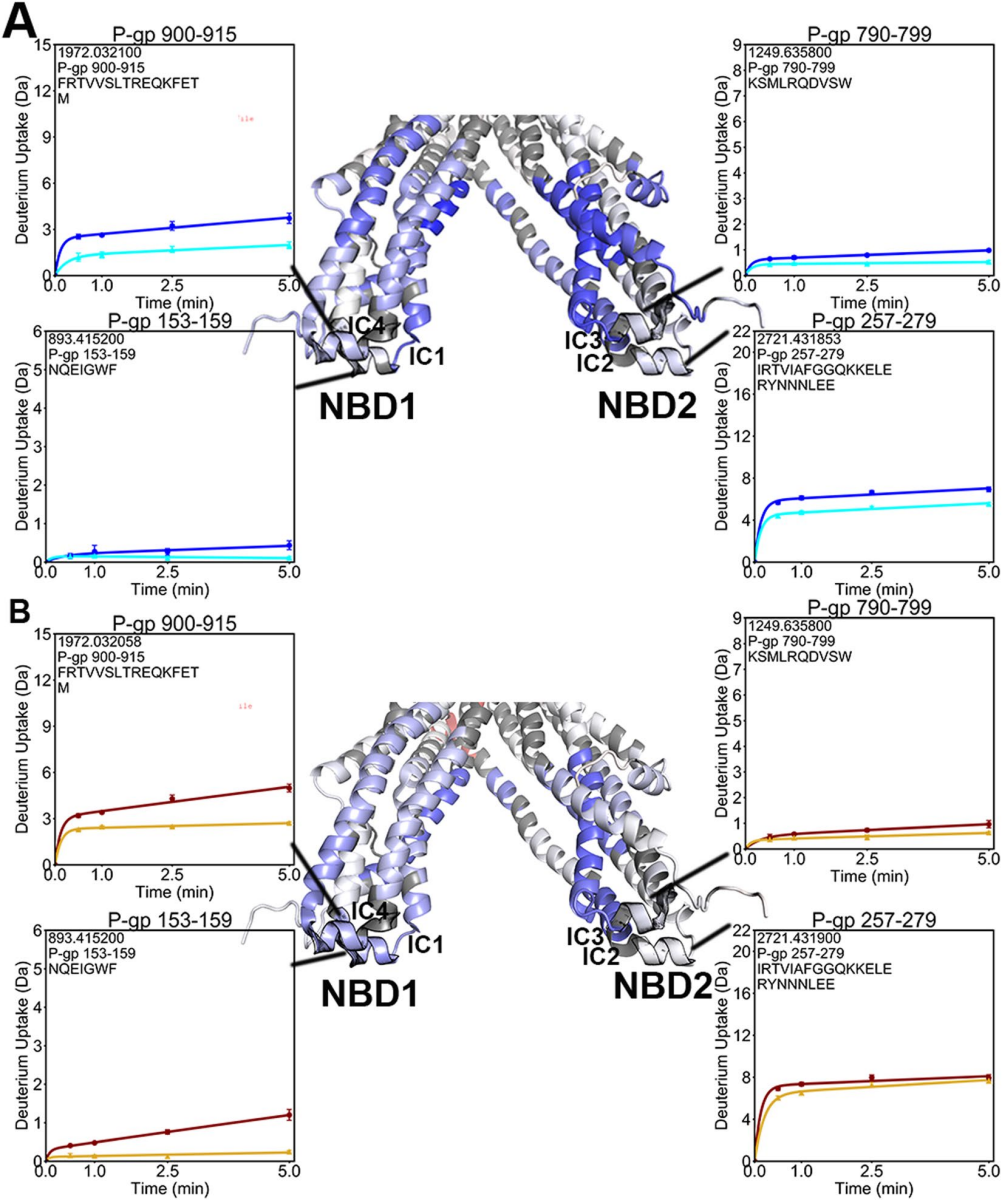
Deuterium uptake plots of the ICHs. (**A**) In the pre-hydrolytic state, ICH4 contacting NBD1 and ICH2 contacting NBD2 showed decreased exchange. (**B)** In the outward-facing state, both ICHs contacting NBD1 showed reduced exchange, while the ICHs at the NBD2 interface did not.

Among the ICHs, one ICH contacting NBD1 (IC4: residues 900–915) and one ICH contacting NBD2 (IC2: residues 257–279) showed reduced exchange in the pre-hydrolytic state (Fig. 6A). In the outward-facing state, both ICHs contacting NBD1 (IC1: residues 153–159, and IC4) showed reduced exchange whereas deuterium incorporation into the ICH pair that interfaces with NBD2 (IC2, and IC3: residues 790–799), was unchanged (Fig. 6B).

### Extracellular domain

**Figure 7:**
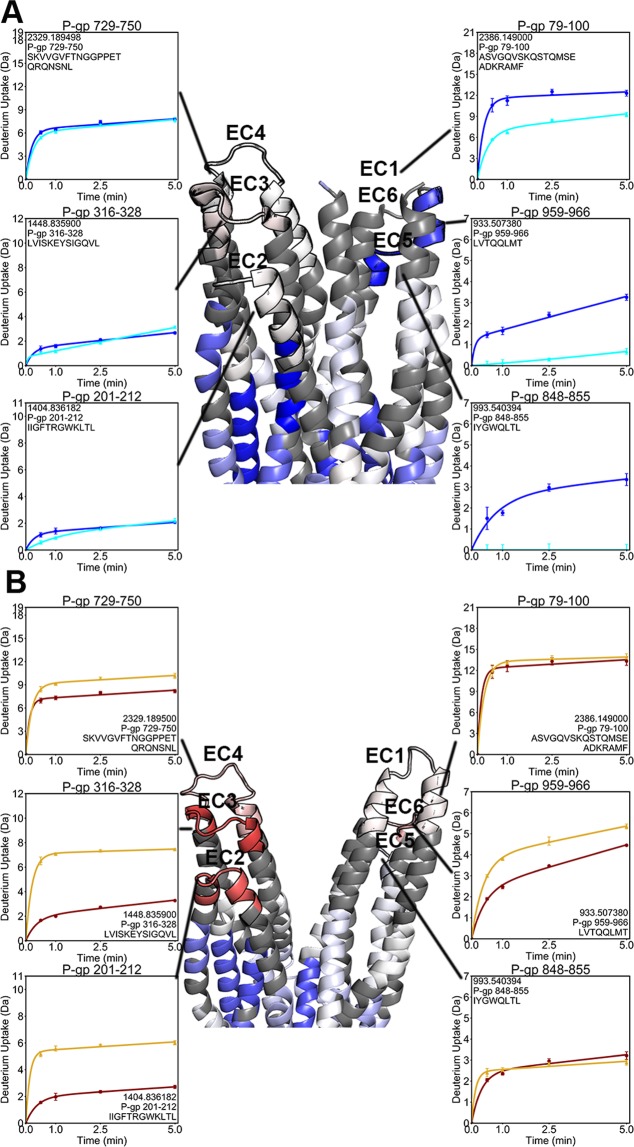
Deuterium uptake plots reveal the dynamics of the EC domain. (**A)** A cryo-EM structure of (E552Q/E1197Q) P-gp obtained under similar experimental conditions provided the best fit to our HDX-MS data^24^. Decreased exchange was evident among ECL1, 5 and 6 in the pre-hydrolytic state. (**B)** In outward-facing P-gp, increased exchange was found among ECL 2, 3, 4 and 6.

The EC domain is comprised of six loops between TMH pairs, and it showed dramatically different changes in the pre-hydrolytic and outward-facing conformations. In the pre-hydrolytic state, EC1 (residues 79–100), EC5 (residues 848–855), and EC6 (residues 959–966) showed reduced uptake (Fig. 7A) While in the outward-facing state, EC2 (residues 201–212), EC3 (residues 316–328), EC4 (residues 729–850) and EC6 incorporated deuterium more rapidly (Fig. 7B).

## Discussion

The high sequence coverage we obtained provided a comprehensive picture of P-gp dynamics in the apo, pre-hydrolytic and outward-facing states. A substantial reduction in deuterium exchange was observed within the NBDs in the pre-hydrolytic and outward-facing states compared to the inward-facing apo state, implying that the two binding sites were occupied by nucleotide in both states (Supp. Fig. 6). These results agree with previous findings that two molecules of ATP were observed bound to P-gp in a cryo-EM structure^24^. In addition, our BLI measurements revealed two binding affinities as did previous studies of Chinese hamster P-gp binding to the non-hydrolyzable ATP analogue ATP-γ-S, which revealed that when one NBD occludes nucleotide with relatively tight affinity (K_D_: 4 µM), the other NBD also remains associated with nucleotide, albeit more weakly (K_D_: 740 µM)^38^.

Consistent with expectations, a region of low exchange due to solvent exclusion by the detergent micelle was represented by a band of complete protection found along the TMHs (Fig. 1A). This region is corresponds to the location of the membrane as determined computationally and by atomic force microscopy of P-gp reconstituted in a phospholipid bilayer^39^. Cryo-EM structures generated using P-gp solubilized with the same detergent employed here also showed the presence of a detergent corona around this region^34,40^.

Two helices (TM2 and TM8), located on opposite sides of the transporter and sandwiched between surrounding TMHs, were the only regions that were protected from exchange outside of the detergent band (Fig. 3). The most likely reason for their low exchange is tight packing with neighboring TMHs, which may restrain dynamic motions necessary for deuterium exchange to occur. These TMHs also did not show any difference in uptake between the three experimental states, suggesting that the dynamics of TM2 and TM8 remain unchanged as P-gp alternates between inward and outward-facing conformations.

Surprisingly, one segment of TM4 (residues 216–223), despite being located along the detergent band, showed relatively high deuterium exchange (Fig. 4). In addition, the (E552Q/E1197Q) mutations in the NBDs were sufficient to reduce deuterium incorporation into this segment, which is over 80 Å away. Exchange within this region was also reduced in the pre-hydrolytic and outward-facing states. These results suggest that this portion of TM4 responds to changes in the NBDs and plays a key role in conformational transitions. Furthermore, it appears that the ATPase inhibitory (E552Q/E1197Q) mutation induces long-distance stabilizing effects even in the absence of ligands. Conformational heterogeneity has previously been noted within this region, where a study of P-gp crystal structures bound to various ligands noted different conformations of this region with the finding that ATPase-stimulating ligands induced a structural change in TM4 between residues 219–243, while binding of ATPase inhibitors resulted in a more rigid structural reorganization^7^.

The polyspecific substrate binding pocket located within the TM domain showed increasing exchange over time (Fig. 4), indicating mobility throughout the TMHs that comprise this pocket. These exchange profiles resulted from amides that were gradually exposed as the transporter sampled a range of conformations over a range of time scales. It is likely that these dynamics contribute to the substrate promiscuity that is a hallmark characteristic of P-gp^41^. These results confirm predictions that the polyspecific binding pocket is highly dynamic, underscoring the mobility of the P-gp molecular machine.

Two ICHs nestle into hydrophobic clefts in each NBD, forming highly conserved interfaces that have been shown to be crucial for P-gp ATPase activity^42,43^. Unlike the similar reductions in exchange observed at the nucleotide binding sites, the two ICH-NBD interfaces displayed asymmetric changes in dynamics. In the pre-hydrolytic state, all NBD regions contacting ICHs decreased exchange (Fig. 5A). In the outward-facing state, every region in NBD1 contacting ICHs decreased exchange while only one portion of NBD2 contacting ICHs decreased exchange (Fig. 5B). At the opposite side of this interface, one ICH contacting each NBD showed lower deuterium exchange in the pre-hydrolytic state (Fig. 6A). All ICH regions that contact NBD1 showed reduced exchange in the outward-facing state, while none of the ICHs contacting NBD2 decreased exchange (Fig. 6B). Similar results demonstrating reduced uptake at the ICH-NBD1 interface in the outward-facing conformation and no change at the ICH-NBD2 site were obtained in another HDX-MS study of P-gp^32^, although coverage of this region was incomplete in the previous work. These results suggest coordinated conformational motions that occur sequentially to move P-gp through a range of conformations.

The EC domain, composed of six loops between TMHs, underwent remarkably different changes in dynamics in the pre-hydrolytic and outward-facing states. We observed that three of the ECLs along the face of the molecule defined by TM10 decreased exchange in the pre-hydrolytic state (Fig. 7A). In contrast, in the outward-facing conformation, three of the ECLs along the side defined by TM4 and one ECL on the TM10 side had increased exchange (Fig. 7B). It is interesting to note that the decrease in exchange in the TMHs following NBD dimerization is similar to the decrease in exchange in the EC domain in the pre-hydrolytic state strongly suggesting the entire molecule is occluded in the pre-hydrolytic state (Supp. Fig. 5). In contrast, our results clearly show that the outward-facing state has increased ECL dynamics. EC domain opening has been observed structurally^10,11^ and EC domain flexibility was suggested by the observed heterogeneity of the EC region in cryo-EM^24^ and by intramolecular distances determined from spin-labeling^15^. Our HDX-MS results imply that the intermediate pre-hydrolytic state is occluded from both the IC and EC environments simultaneously, and they suggest a mechanism by which both substrate entry and exit points are occluded, preventing temporary channel formation during transport.

## Conclusion

Our results provided unprecedented HDX-MS coverage of P-gp, enabling a comprehensive view of dynamics in three distinct conformational states. Decreased exchange in the NBDs reflects the NBD dimerization that occurs following nucleotide binding. The asymmetric perturbations at the two ICH-NBD interfaces suggest that the two pseudosymmetric halves of the molecule function differently from one another with regard to dynamics. However, similar nucleotide binding kinetics measured for wild-type and (E552Q/E1197Q) P-gp indicate that these perturbations do not impact overall nucleotide affinity. High dynamics of the substrate-binding pocket in the TM domain implies conformational flexibility that likely promotes substrate promiscuity, while lower than expected dynamics of the linker indicate that the linker may possess some secondary structure. The most striking observation, however, was the differences in the deuterium uptake of the ECLs between the apo, pre-hydrolytic, and outward-facing states. In the pre-hydrolytic state, the dynamics in this region decreased while the same region increased dynamics in the outward-facing state when compared to apo P-gp. These findings indicate a mechanism which prevents the transporter from behaving as a channel during the intermediate transition between the inward-facing and outward-facing states. Our findings suggest that this occluded conformation occurs at the ATP bound pre-hydrolytic stage of transport, and they characterize P-gp as a highly dynamic machine undergoing multiple correlated motions to drive substrate translocation while avoiding leakage.

## Materials and Methods

### P-gp expression and purification

Codon-optimized murine P-gp (Genbank: JF834158) was expressed in *Pichia pastoris* and purified as previously described^8^. Size-exclusion chromatography (SEC; Superdex 200 16/60, GE Healthcare) was performed with buffer containing 20 mM HEPES pH 7.5, 100 mM NaCl, 0.035% β-DDM, 0.01% sodium cholate, and 0.2 mM TCEP. Fractions were pooled and stored at 80 °C for HDXMS analysis.

### HDX-MS analysis of P-gp

HDX-MS measurements were made using a Synapt G2Si system (Waters Corporation). Deuterium exchange reactions were carried out by a Leap HDX PAL autosampler (Leap Technologies, Carrboro, NC). Deuterated buffer was prepared by lyophilizing 10 mL of 20 mM HEPES pH 7.5, 100 mM NaCl. Lyophilized buffer was resuspended in 10 mL 99.96% D_2_O immediately before use, to which was added powdered β-DDM to a final concentration of 0.01%. For measurements of the outward-facing state, the following reagents were added to P-gp samples and D_2_O buffer at final concentrations: 5 mM MgSO_4_, 2 mM sodium orthovanadate and 2 mM ATP. For measurements of the pre-hydrolytic state, 5 mM MgSO_4_ and 10 mM ATP were added to (E552Q/E1197Q) P-gp samples and D_2_O buffer, as these conditions were found to induce a NBD-dimerized conformation in a majority of particles observed by cryo-EM^24^. In the apo, pre-hydrolytic, and outward-facing states, control solutions were added to protein samples and D_2_O buffer in order to account for buffer dilution and the addition of H_2_O. Each deuterium exchange time point (0 min, 30 sec, 1 min, 2.5 min, 5 min) was measured in triplicate. For each measurement, 4 µL of protein was mixed with 36 µL of D_2_O buffer at 25 °C. Deuterium exchange was quenched by combining 35 µL of the deuterated sample with 65 µL of 0.1% formic acid and 3 M guanidinium-HCl for 1 min at 1 °C. The quenched sample was then injected in a 50 µL sample loop and digested by an inline pepsin column (Pierce, Inc.) at 15 °C. Optimization revealed that ideal sequence coverage was obtained by using a flow rate of 400 µL/sec to capture the resulting peptides on a BEH C4 Vanguard precolumn. Peptides were then separated by analytical chromatography (Acquity UPLC BEH C4, 1.7 uM, 1.0 × 50 mm, Waters Corporation) using 7−85% acetonitrile in 0.1% formic acid over 7.5 min, and then analyzed on a Waters Synapt G2Si quadrupole time-of-flight mass spectrometer following electrospray injection.

Data were collected in Mobility, ESI + mode, mass acquisition range of 200−2000 (m/z), scan time 0.4 s. Continuous lock mass correction was performed using infusion of leu-enkephalin (m/z = 556.277) every 30 seconds (mass accuracy of 1 ppm for calibration standard). For peptide identification, data were instead collected in MS^E^ (mobility ESI+) mode. Peptides masses were identified following triplicate analysis of 10 µM P-gp, and the data were analyzed using PLGS 2.5 (Waters Corporation). Peptides masses were identified using a minimum number of 250 ion counts for low energy peptides and 50 ion counts for their fragment ions. The following parameters were used to filter peptide sequence matches: minimum products per amino acid of 0.2, minimum score of 7, maximum MH+ error of 5 ppm, and a retention time RSD of 5%, and the peptides had to be present in two of the three ID runs collected. After identification in PLGS, peptides were analyzed in DynamX 3.0 (Waters Corporation). Deuterium uptake for each peptide was calculated by comparing the centroids of the mass envelopes of the deuterated samples with the undeuterated controls. Back-exchange correction factors were applied as previously reported^44^ The Y-axis limit for each plot reflects the total number of amides within the peptide that can possible exchange. Each plot includes the peptide MH+ value, sequence, and sequential residue numbering.

### Binding kinetics

Analysis of binding kinetics were carried out using a ForteBio K2 at 30 °C in P-gp storage buffer. Wild-type and (E552Q/E1197Q) P-gp were biotinylated using EZ-Link NHS-PEG4-Biotin (Thermo Scientific) at a ratio of 1:1. Streptavidin coated biosensors were pre-hydrated in P-gp storage buffer for 15 min and then transferred to microplate wells containing biotinylated P-gp at a concentration of 6 µg/mL for immobilization. Unbound streptavidin molecules were blocked by incubation in 10 µg/mL biocytin for 60 sec, and biosensors were then washed an additional 60 sec in storage buffer. Baseline BLI measurements were carried out for 90 sec in storage buffer. Association rates were measured by transferring the biosensors to wells supplemented with Mg^+2^ (or Mg^+2^ and Na_3_VO_4_) and nucleotide for 60 sec. Dissociation was then measured for 180 sec by transferring the biosensors back to the wells used for baseline recording. In the wells used for association measurements, concentrations of Mg^+2^ (or Mg^+2^ and VO_4_^−3^) and nucleotide were maintained at a 1:1 ratio. Signal due to changing Mg^+2^ and VO_4_^−3^ concentrations was subtracted by referencing against buffer supplemented with only Mg^+2^ and VO_4_^−3^ without nucleotide. Non-specific binding of ATP to the biosensor surface was subtracted by referencing against biosensors with no P-gp immobilized. Because high concentrations of nucleotide were necessary to obtain a signal, a control experiment was carried out using biotinylated bovine serum albumin to ensure there was no non-specific binding of nucleotide to the immobilized protein. Kinetics data were fitted to a two binding-site model using ForteBio Data Analysis 11.0 and were plotted with Kaleidagraph.

### ATPase assay

P-gp samples were diluted to give 1 µg per 30 µL in P-gp storage buffer supplemented with 5 mM MgSO_4_ and 0.1 mg/mL *e*. *coli*. polar lipids. Verapamil-HCl stock solution was made using the same buffer, and P-gp was incubated with verapamil-HCl or buffer for 15 min on ice. Protein samples (30 µL) and reaction buffer containing 50 mM ATP (1.6 µL) were then transferred to separate wells on a 96-well plate held at 4 °C on a thermocycler. The P-gp samples were added to wells containing ATP using a multichannel pipette and the plate was cycled to 30 °C for 3 min of hydrolysis, then brought to 80 °C for 15 sec to inactivate P-gp and held at 4 °C. Inorganic phosphate liberated by hydrolysis was measured by adding 30 µL of the samples to 150 µL of developing solution prepared by mixing 0.525 g ammonium molybdate in 12.5 mL of 4 M HCl with 17 mg malachite green in 37.5 mL of deionized H_2_O and adding 0.1% (v/v) Triton-x 100 to activate. Absorbance was measured at 595 nM on a PerkinElmer 2030 plate reader after 5 min.

## Supplementary information

Supplementary information
