# Supplementary material for: Dynamics of ABC Transporter P-glycoprotein in Three Conformational States
Source: Sci Rep. 2019 Oct 22;9:15092. doi: 10.1038/s41598-019-50578-2 (PMC6805939; doi:10.1038/s41598-019-50578-2)
Supplement: Supplementary file 1 — Supplementary information [file 41598_2019_50578_MOESM1_ESM.docx]

**
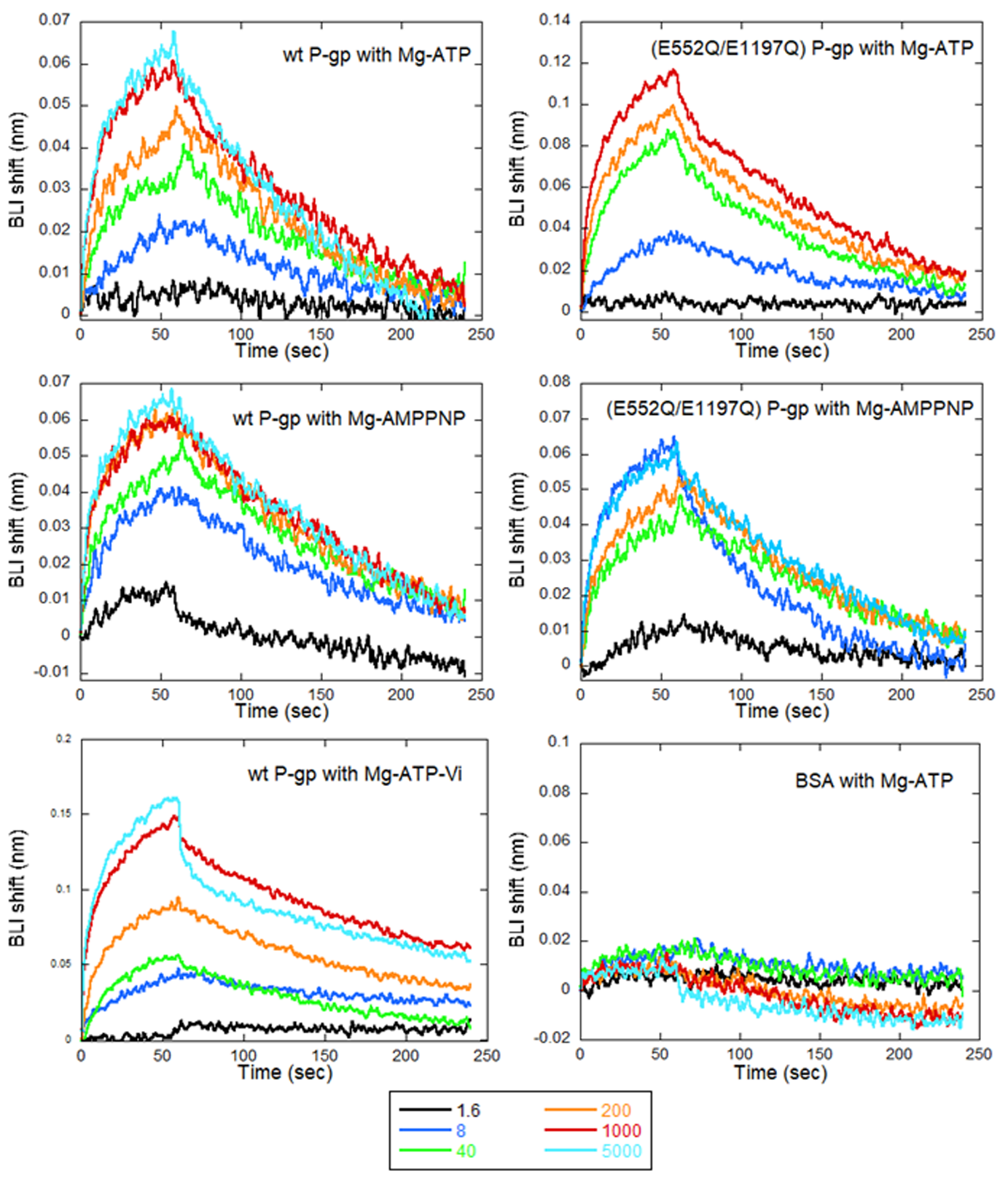
**

**Supplementary Figure 1.** Biolayer Interferometry (BLI) plots for wild-type and (E551Q/E1197Q) P-gp binding to ATP and AMPPNP. Concentrations are shown in μM.


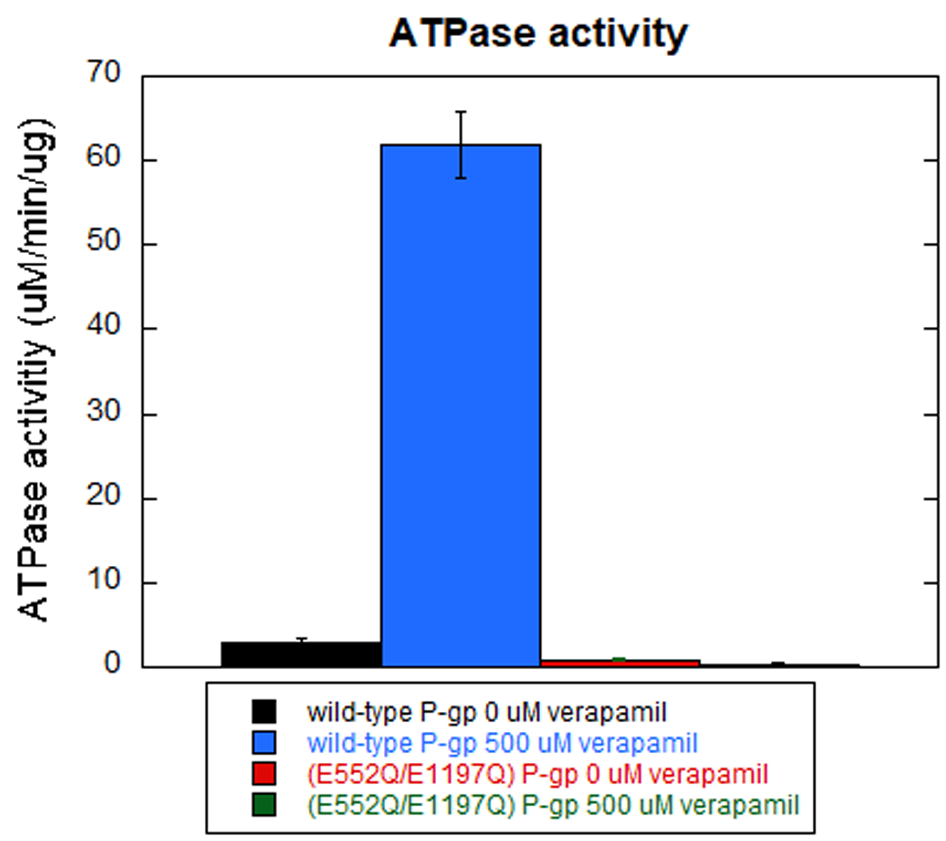


**Supplementary Figure 2.** ATPase activity of wild-type P-gp was enhanced by the substrate transport verapamil, while (E552Q/E1197Q) P-gp was insensitive to verapamil.

**
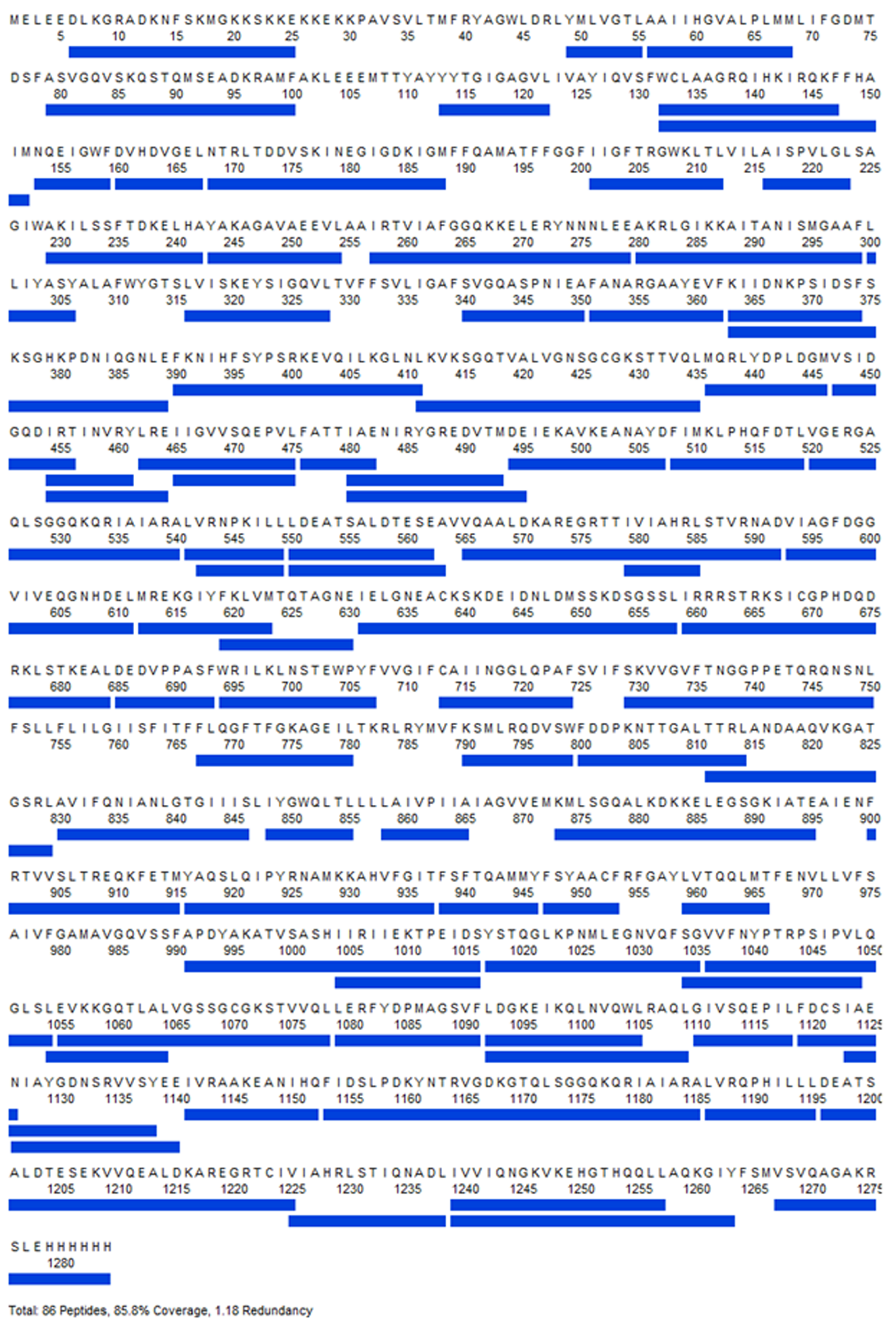
**

**Supplementary Figure 3.** HDXMS sequence coverage of P-gp. A total of 86 peptides were identified, representing 85.8% of the P-gp sequence.

**
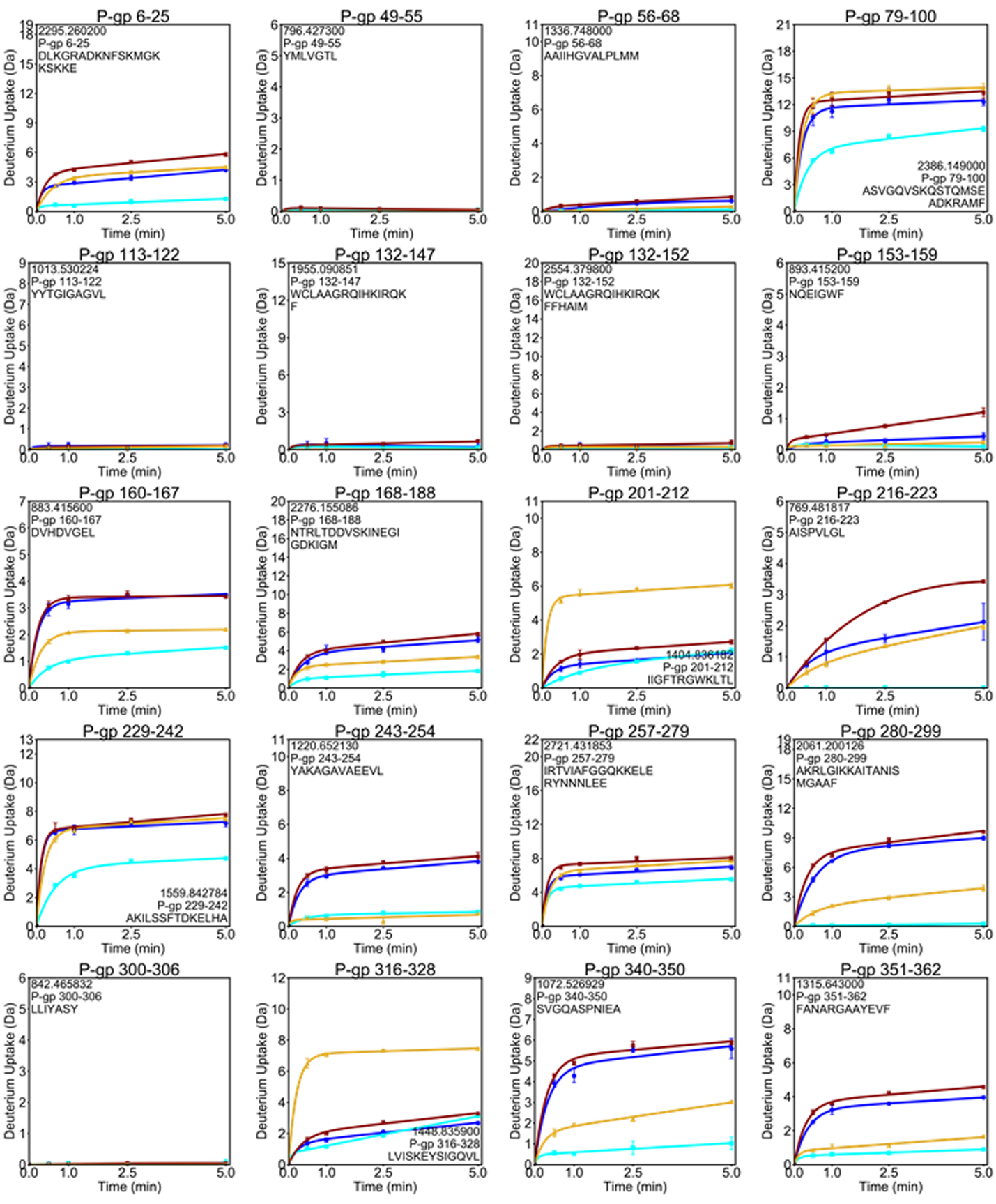
**

**
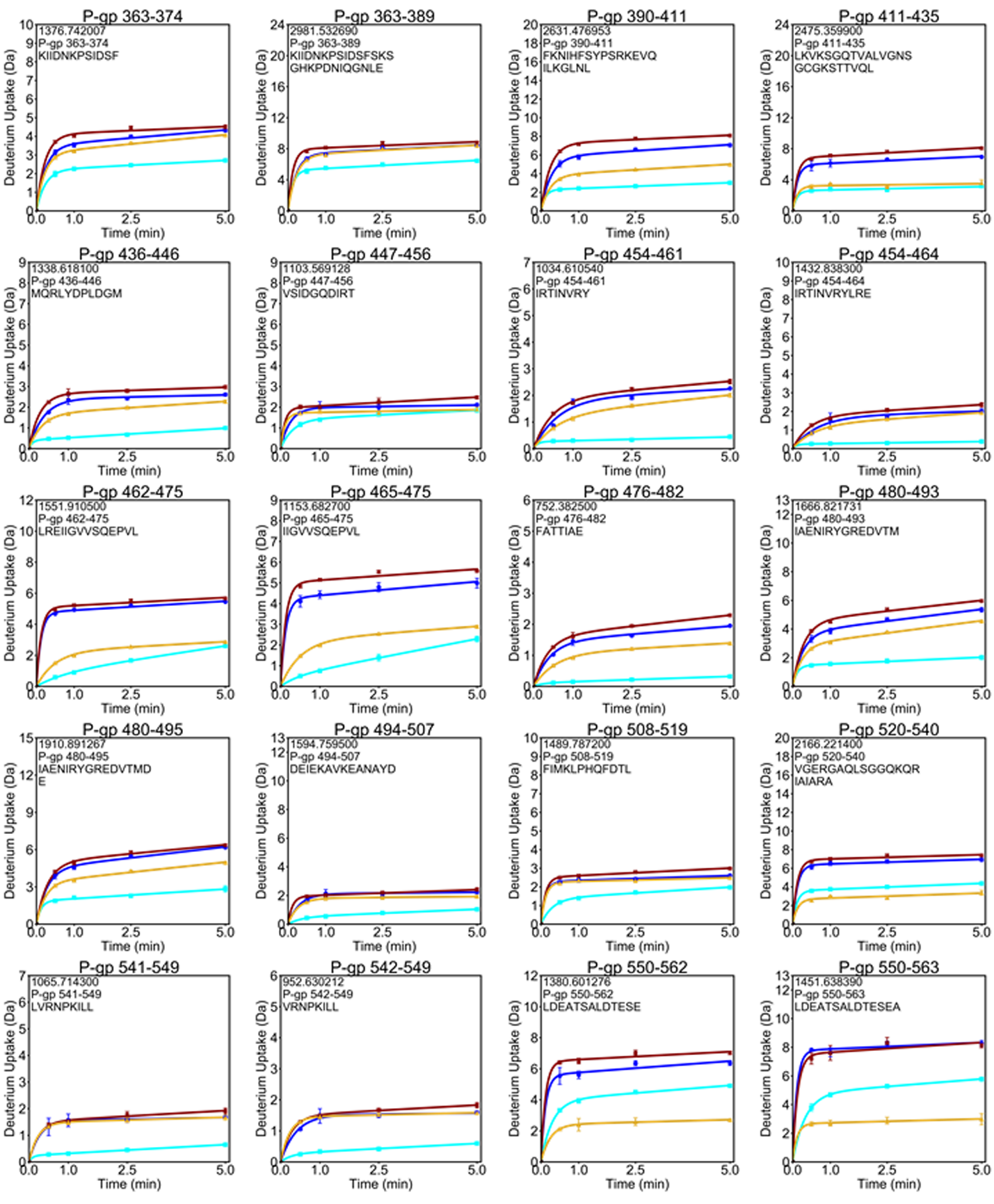
**

**
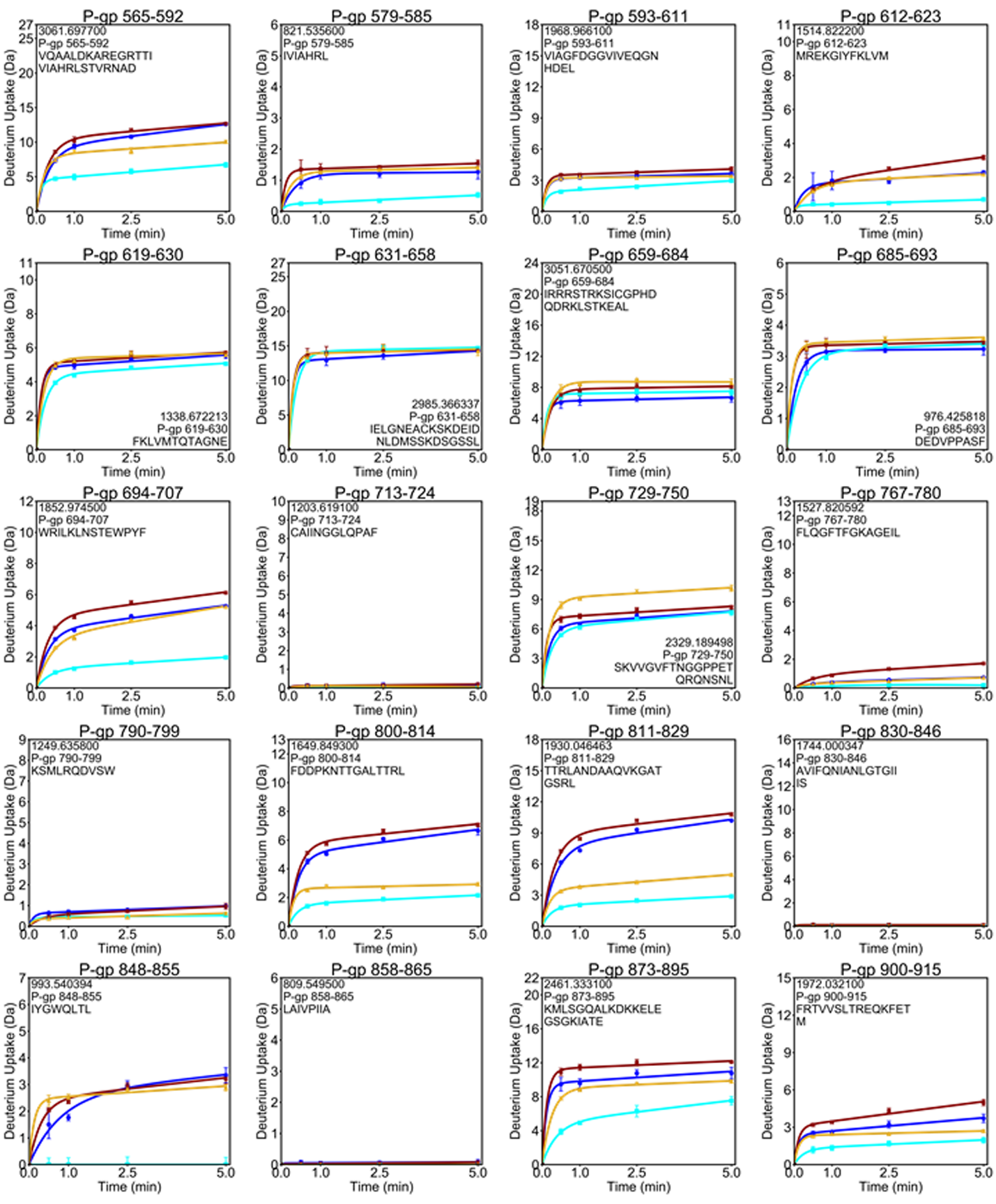
**

**
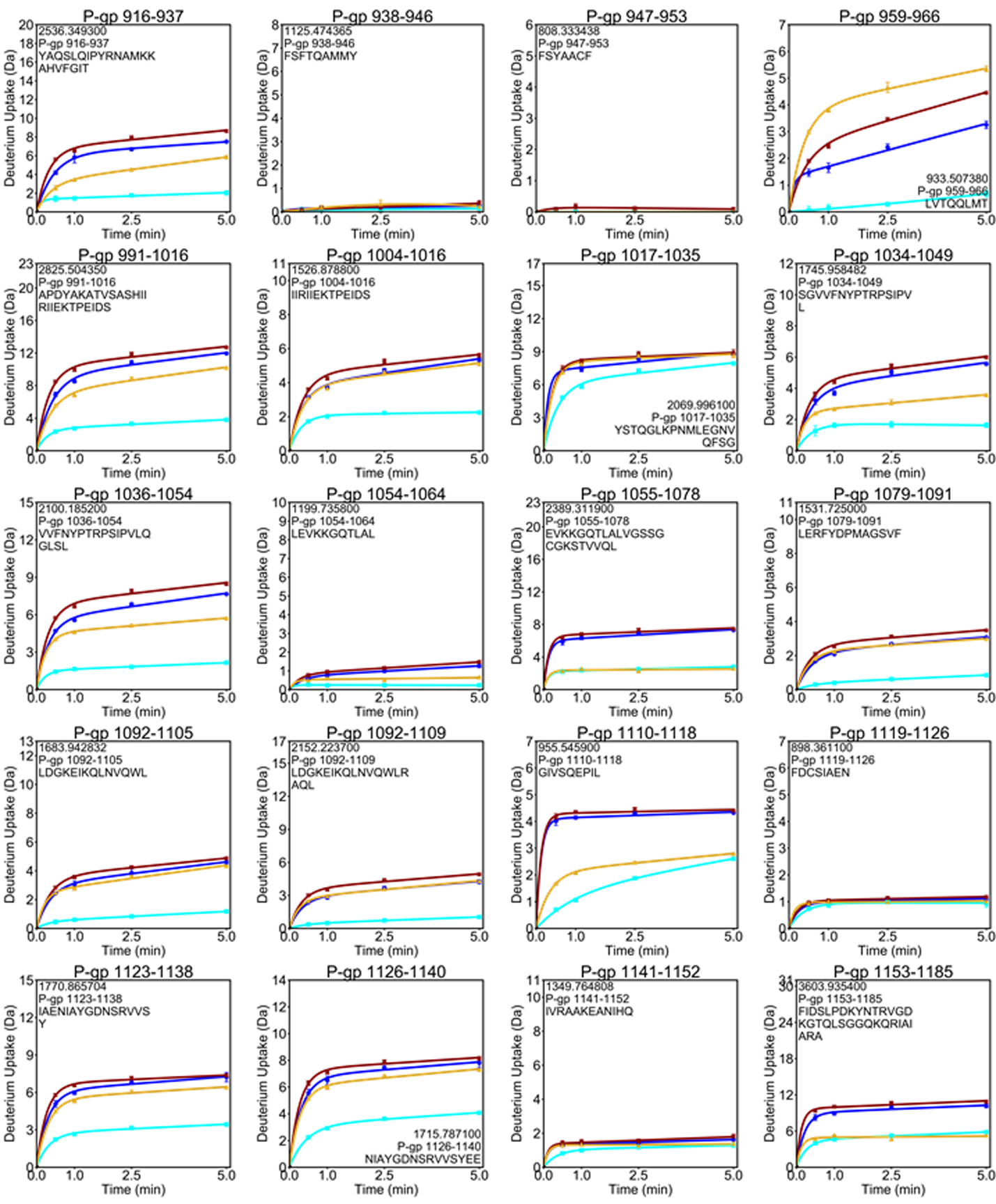
**

**
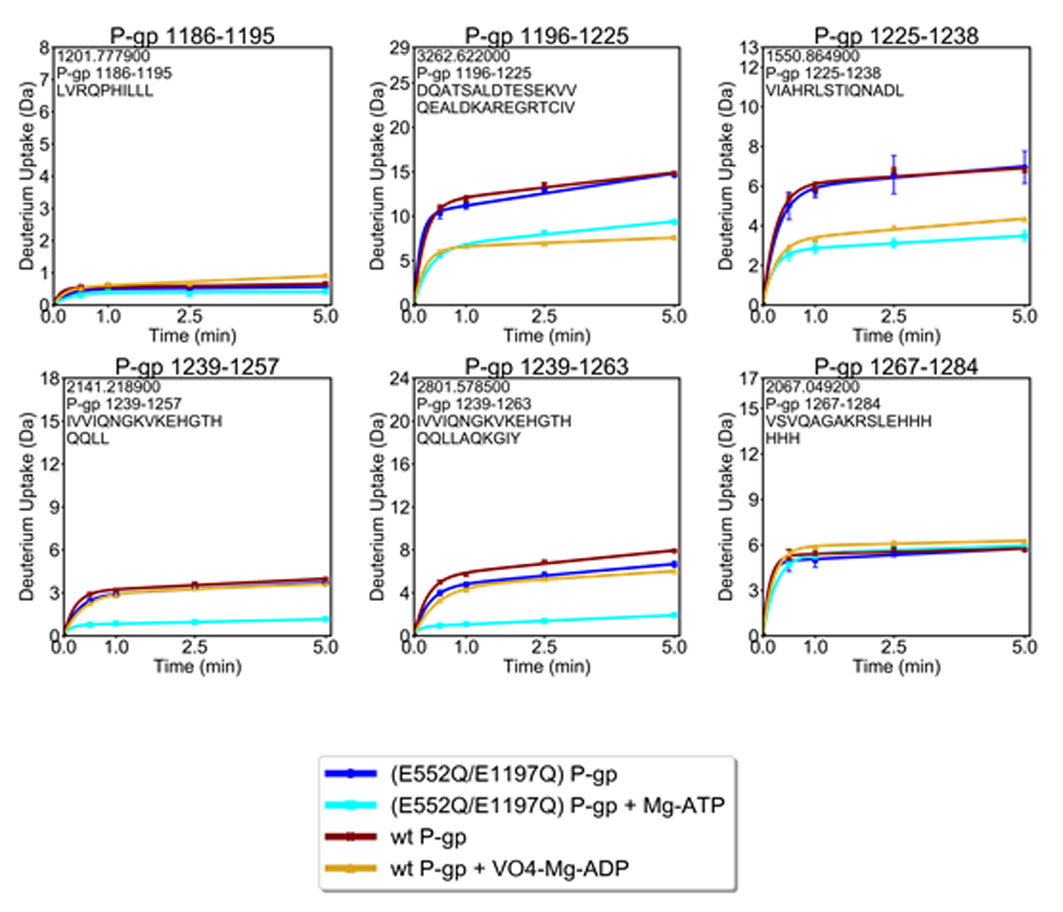
**

**Supplementary Figure 4.** Deuterium uptake plots for all peptides are shown.

**
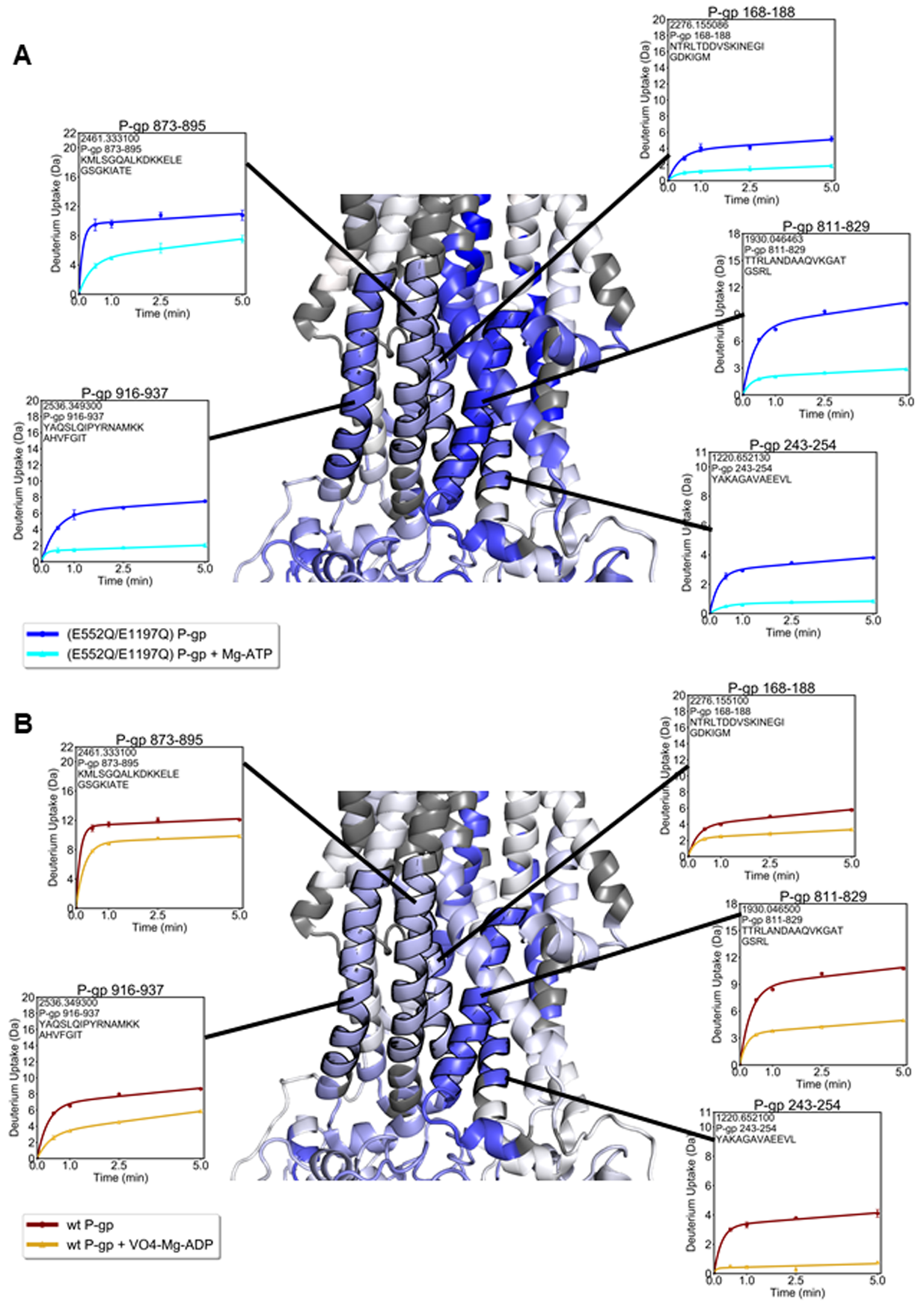
**

**Supplementary Figure 5.** Inner TM helices which group together following NBD dimerization all decreased exchange in both pre-hydrolytic **(A)** and outward-facing **(B)** states.

**
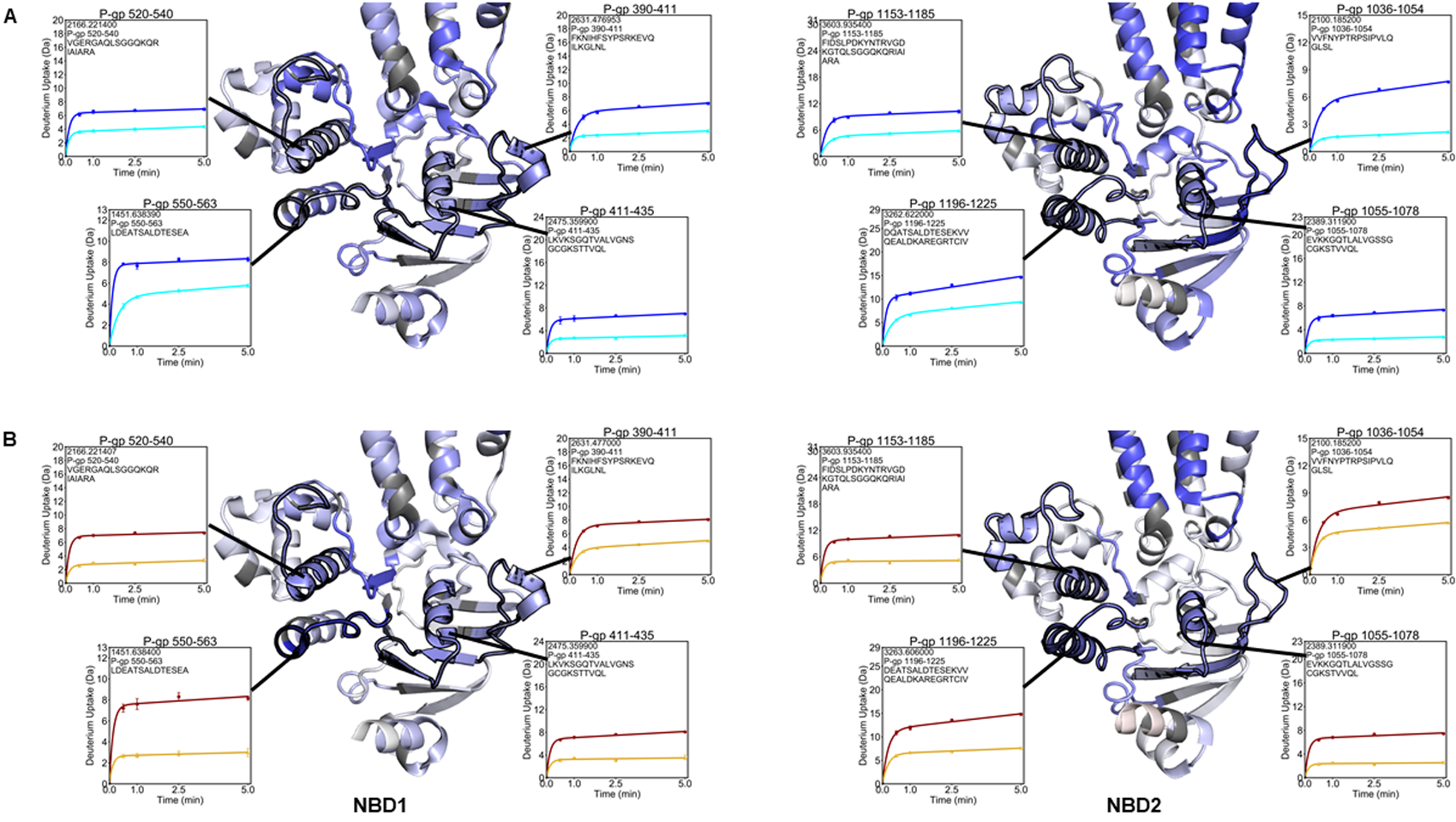
**

**Supplementary Figure 6.** Conserved ABC transporter motifs and regions which contact nucleotide from both NBDs decreased exchange in the pre-hydrolytic and outward-facing states.
